# The potential of *Mitragyna speciosa* leaves as a natural source of antioxidants for disease prevention

**DOI:** 10.1515/jib-2023-0030

**Published:** 2024-09-17

**Authors:** Ihsanul Arief, Gagus Ketut Sunnardianto, Syahrul Khairi, Wahyu Dita Saputri

**Affiliations:** Research Center for Quantum Physics, 599846 National Research and Innovation Agency (BRIN), South Tangerang 15314, Indonesia; Akademi Farmasi Yarsi Pontianak, Pontianak 78232, Indonesia; School of Materials Science and Engineering, Nanyang Technological University, 50 Nanyang Avenue, Singapore 639798, Singapore; Department of Chemical Engineering, Faculty of Engineering, Universitas Tanjungpura, Pontianak 78124, Indonesia

**Keywords:** *Mitragyna speciosa*, antioxidant, virtual screening, ADMET, molecular dynamics

## Abstract

*Mitragyna speciosa* is famous for its addictive effect. On the other hand, this plant has good potential as an antioxidant agent, and so far, it was not explicitly explained what the most contributing compound in the leaves to that activity is. This study has been conducted using several computational methods to determine which compounds are the most active in interacting with cytochrome P450, myeloperoxidase, and NADPH oxidase proteins. First, virtual screening was carried out based on molecular docking, followed by profiling the properties of adsorption, distribution, metabolism, excretion, and toxicity (ADMET); the second one is the molecular dynamics (MD) simulations for 100 ns. The virtual screening results showed that three compounds acted as inhibitors for each protein: (-)-epicatechin, sitogluside, and corynoxeine. The ADMET profiles of the three compounds exhibit good drug ability and toxicity. The trajectories study from MD simulations predicts that the complexes of these three compounds with their respective target proteins are stable. Furthermore, these compounds identified in this computational study can be a potential guide for future experiments aimed at assessing the antioxidant properties through *in vitro* testing.

## 1 Introduction

The current pollution level increases are directly proportional to the number of diseases associated with oxidative stress. Therefore, the urgency of discovering antioxidants is currently needed. In laboratory tests as *in vitro* methods, antioxidant agents must stabilize the radical 2,2-diphenyl-1-picrylhydrazyl (DPPH). While in the human body, an antioxidant agent could have various modes of action, and it can inhibit the protein cytochrome P450, myeloperoxidase, or NADPH oxidase [1]. Cytochrome P450 (CP450) enzymes are expressed throughout the body and are essential to human physiology. They are involved in the metabolism of drugs and xenobiotics and the biosynthesis of endogenous molecules. Nevertheless, during the CP450 catalytic cycle, reactive oxygen species (ROS) can be produced by uncoupling the enzymatic cycle [2]. On the other hands, HOCl can be produced by the interaction of chloride and H_2_O_2_ when myeloperoxidase (MP) is activated. Additionally, MP causes oxidative stress by stimulating the generation ROS and reactive nitrogen species (RNS), influencing the polarisation and signalling pathways associated with inflammation in neutrophils and microglia [3]. The production of reactive oxygen species (ROS), principally superoxide anion (O_2_^·−^), though H_2_O_2_ can also be produced, is attributed to the NADPH oxidase (NO), an electron-transporting membrane protein. Increased ROS cause oxidative stress, which has been linked to numerous inflammatory and degenerative diseases [4]. One of the most prevalent diseases associated with oxidants is cancer, which has become the second leading cause of death. Antioxidants obtained from natural resources are believed to be one of the ways to prevent the disease [5].

Kratom (*Mitragyna speciosa*) is a tropical plant commonly found in Indonesia. The rampant increase in kratom consumption is also marked by the many ordinary plant farmers turning to kratom farmers because this plant’s cultivation is considered more economically promising. Moreover, it was recorded that until July 2021, kratom exports to the United States had reached 400 tons. Even so, this plant has been becoming polemic in Indonesia due to its extract being categorized as an addictive substance [6]. Despite that, this plant has emerged as a potential source of natural compounds with diverse biological activities, including an antioxidant agent, as reported by Firmansyah et al. [7]. Fifty-four compounds were found in kratom, and 17 were successfully isolated from the leaves [8, 9]. However, among the various compounds in kratom leaves, neither *in silico* nor *in vitro* analysis currently exists that elucidates which compound exerts the most significant antioxidant effect.

*In silico* methods have been implemented in many fields. In drug discovery, it was used as a tools to search promising lead compound, one of the technique is docking [10, 11]. Another recent *in silico* using was in drug management, in example for personalized medicine [12]. *In silico* approach is the focus of this study, and one of the methods of this approach is Virtual screening (VS). This method has become one of the computational studies widely used to define the most compromising molecule to a particular bioactivity from natural products [13–17]. There are two approaches in VS, ligand-based (LBVS) and structure-based (SBVS) virtual screening, which have been more prevalent recently [18]. SBVS is based on molecular docking, in which the interaction between protein and ligand can be studied further through the energy profiles of the highest occupied molecular orbital (HOMO) and lowest unoccupied molecular orbital (LUMO) because the interaction occurs between the two orbitals [18].

Besides activity, other essential properties in drug discovery are drug ability and toxicity. The drug ability of a compound determines its fate as it passes through the gastrointestinal tract until it reaches the expected target protein. This property is primarily expected to be unchanged in antioxidant compounds which are generally reactive. Drug ability is generally described through adsorption, distribution, metabolism, and excretion (ADME) profiles [19]. At the same time, the toxicity of a compound is also important to study in several organs, such as the liver, cytochromes, and its potential as a carcinogen [20, 21]. As a form of initial screening, several platforms are currently available to predict the properties of ADME and the toxicity of compounds, i.e., admetSAR, ProTox-II, DrugMint, FAF-Drugs4, DLF, SwissADME, and ADMETLab [21].

For further study, the docked protein-ligand complexes from SBVS are then studied via molecular dynamics (MD) simulations to ensure their stability [22]. The MD simulations have been applied to the antioxidant activities studies 23], [24], [25. Several aspects neglected in docking are involved using the MD simulation, such as the presence of water and protein molecules, which are set to move dynamically [26, 27]. This method brings more accurate data because the molecular setup is more similar to the actual situation.

In this study, structure-based virtual screening (SBVS) was executed to cytochrome P450, myeloperoxidase, or NADPH oxidase proteins associated with antioxidant activity and followed by frontier molecular orbital profiling. Hence, the ADMET properties of selected molecules were also predicted, and the stability of protein-ligand complexes was analyzed with molecular dynamics (MD) simulation for 100 ns. It is important to emphasize that this study does not encompass *in-vitro* testing. However, the validity of these methods can be evaluated by comparing the results with prior research demonstrating the reliability and validity of similar computational approaches 28], [29], [30.

## 2 Materials and methods

### 2.1 Protein and ligand preparation

The targeted proteins used in this study were obtained from the RCSB database (https://www.rcsb.org/) with a PDB ID of 1OG5 (Cytochrome P450, CP450) and 1DNU (human myeloperoxidase, MP) and 2CDU (NADPH oxidase, NO). All the proteins were separated from non-standard residues using the DockPrep function in Chimera 1.16 [31] and then charge-assigned using the Gasteiger methods [32]. The ligands studied in this work were retrieved from the PubChem database (https://pubchem.ncbi.nlm.nih.gov/) with the CID of 65080, 72276, 94160, 120678, 441975, 3000341, 3034396, 3037629, 5742590, 9930064, 10475115, 10948612, 11726520, 15560576, 44301524, 44568160, and 102183193. The ligands were also prepared using DockPrep in Chimera 1.16, including adding hydrogen atoms and structural minimization using AM1-BCC methods [33, 34].

### 2.2 Virtual screening

Structure-based virtual screening was performed using Autodock Vina [35], implemented in the PyRx 0.8 package [36]. The position and the size of grid boxes were adjusted by the natural ligand (for COX-1) and the literature (for CP450 and MP) [37]. The native ligands used were diclofenac to COX-1, fluorouracil to CP450, and melatonin to MP. The average size of grid boxes was around 20 × 20 × 20 Å, and the exhaustiveness was set to 20. The resulting protein-ligand complexes were evaluated based on the binding affinity values and the type of interactions using the Biovia Discovery Studio Visualizer [38]. The chosen ligand had the most comparable interactions with the positive controls.

### 2.3 Calculation of frontier molecular orbital

The energy levels of the highest occupied molecular orbital (HOMO) and the lowest unoccupied molecular orbital (LUMO) from the ligands and interacting amino acid residues were calculated using the Gaussian 16W package [39]. All the molecular structures were optimized using the DFT level of theory, the B3LYP functional, and the 6-311G(d,p) basis set [40].

### 2.4 Prediction of ADMET properties

The ADME properties of selected ligands were predicted using the SwissADME server (http://www.swissadme.ch/index.php) by submitting the SMILES codes of the ligands [41]. The collected data included GI absorption, BBB permeant, CYP inhibition, Lipinski violation, synthetic accessibility, and bioavailability. At the same time, the toxicity profiles of the selected ligands were computed by the ProTox-II server (https://tox-new.charite.de/protox_II/index.php?site=home) [42].

### 2.5 Molecular dynamics simulation

In the SBVS step, we choose the three protein-ligand complexes that show the most similar interaction to the native ligand and have a remarkable binding affinity. To determine the stability of the docked complexes, we used molecular dynamics (MD) simulation with the Amber22 package [43, 44]. The proteins and ligands were prepared before the simulation using the Antechamber module [34]. The complexes were then minimized for 5 ns using the steepest gradient descent methods and equilibrated for 10 ns, with the temperature adjusted to 300 K using a Langevin thermostat and the density adjusted to 1 atm applying Berendsen barostat in the SHAKE algorithm [45]. Once the complexes were equilibrated, they were subjected to a simulation without restraint for 100 ns, employing a timestep of 2 fs. The simulation employed a Langevin thermostat and a Berendsen barostat, consistent with the equilibration step. The stability of complexes was analyzed based on the profile of root mean square of deviation (RMSD), root mean square of fluctuation (RMSF), the radius of gyration (RoG), hydrogen bond, and Molecular Mechanics Poisson-Boltzmann Surface Area (MMPBSA) free energy using AmberTools for the last 30 ns of the simulation [46].

## 3 Result and discussion

### 3.1 Virtual screening

The result of the virtual screening is shown in Table 1. The most influential parameter in molecular docking based methods was the binding affinity, where the compound that shows the most negative binding affinity to the targeted protein was assumed to have the strongest affinity to the protein in the experimental study [47]. As the pharmacological activity of a ligand was not only dependent on its binding affinity, we also considered the interaction types of the ligands to the targeted proteins. To proceed with further analysis, we selected the ligand that exhibited the interactions most similar to those of the positive control. In the docking-based methods, a protocol is considered valid if the protocol could result in similar interactions compared to the experimental or the root mean square of deviation (RMSD) was below 2 Å [10]. For the CP450, fluorouracil was used as the positive-control ligand and showed a similar interaction to warfarin as the native ligand [48]. The same result was found for MP protein, where we used melatonin as the control ligand and showed the hydrogen bond to HIS336, similar to the experimental result [49].

**Table 1:** The result of virtual screening against CP450, MP, NO proteins, in which Fluorouracil, Melatonin, and Dextromethorphan are the positive control, respectively.

Ligand	CID	Binding affinity (kcal/mol)		
		CP450	MP	NO
Fluorouracil		−4.6		
Melatonin			−8.1	
Dextromethorpon				−6.3
(-)-Epicatechin	72276	**−8.3**	−9.3	−7.8
Sitogluside	5742590	−5.9	**−9.0**	−4.9
Corynoxeine	44568160	−8.5	−7.7	**−7.4**
7-Epi-Vogeloside	102183193	−7.8	−8.5	−7.1
7-Hydroxymitragynine	44301524	−7.1	−8.8	−6.7
Ajmalicine	441975	−9.5	−9.0	−8.5
Corynantheidine	3000341	−7.8	−8.2	−6.4
Corynoxine	10475115	−7.5	−7.9	−5.5
Isocorynantheidine	10948612	−8.6	−9.0	−7.0
Isomitraphylline	11726520	−8.2	−8.4	−7.0
Mitragynine	3034396	−7.9	−8.2	−6.5
Mitraphylline	94160	−8.3	−8.8	−7.1
Paynantheine	3037629	−7.9	−8.1	−8.3
Phenyl beta-D-glucopyranoside	65080	−6.9	−8.0	−6.5
Quinovic acid	120678	−8.1	−7.9	−4.7
Roseoside	9930064	−7.8	−8.6	−6.4
Speciociliatine	15560576	−7.6	−8.5	−6.9

*The bold type number shows the selected ligand from each of the protein.

**Figure 1:**
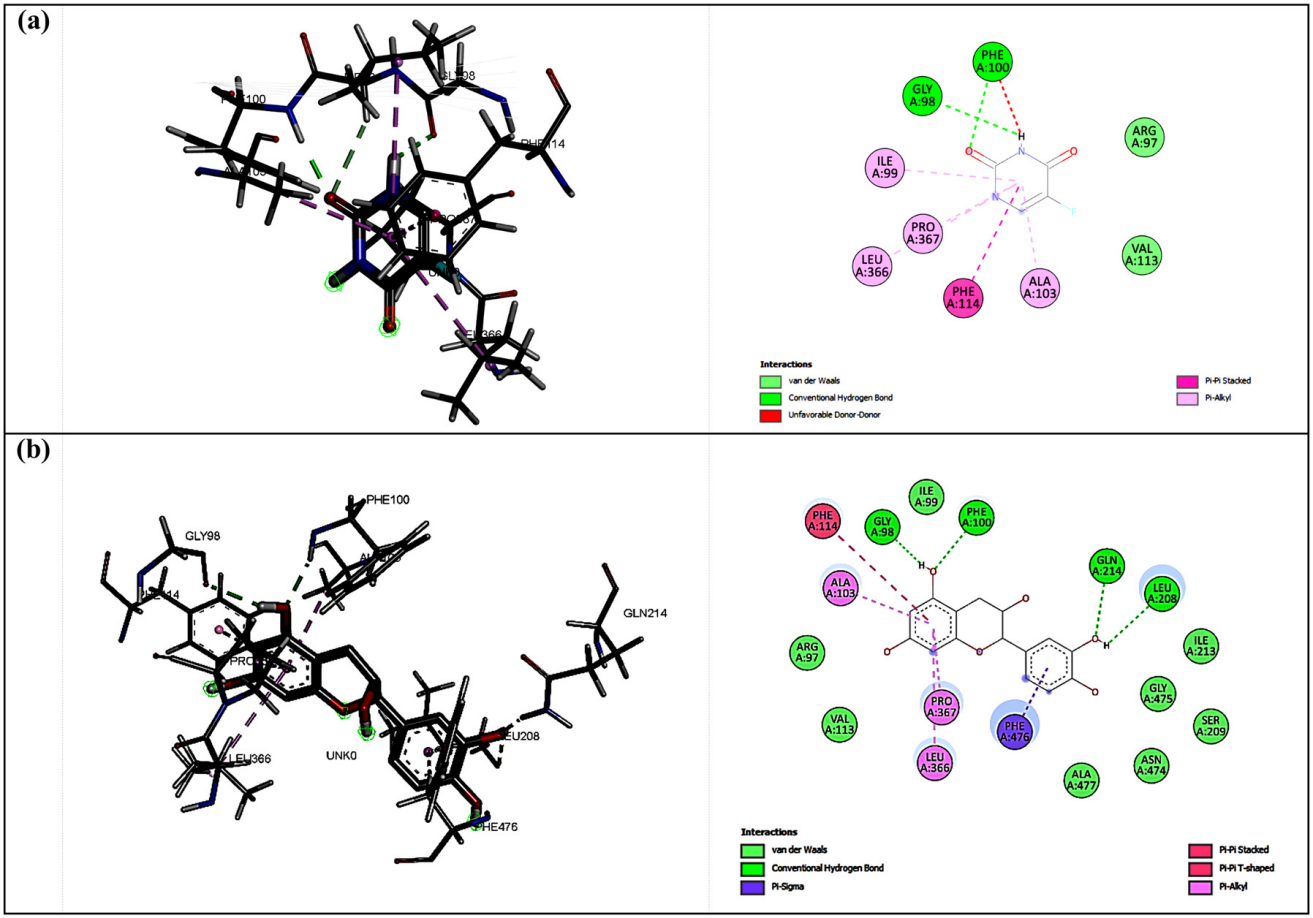
The interactions between fluorouracil (a) and (-)-epicatechin (b) with CP450 protein.

For the first protein, CP450, the screened ligand was (-)-epicatechin (CID: 72276) since it had the most similar interaction to fluorouracil as the positive control. The essential residue inhibited by the antioxidant agent in CP450 protein, especially fluorouracil, was GLY90 and PHE100 [37]. Moreover, (-)-epicatechin shows more hydrogen bond interactions, including toward LEU208 and GLN214, as shown in Figure 1. Adding hydrogen bonds leads to a stronger binding affinity, while the CP450-fluorouracil complex had −4.6 kcal/mol of binding affinity, and the CP450-epicatechin complex had −8.3 kcal/mol.

**Figure 2:**
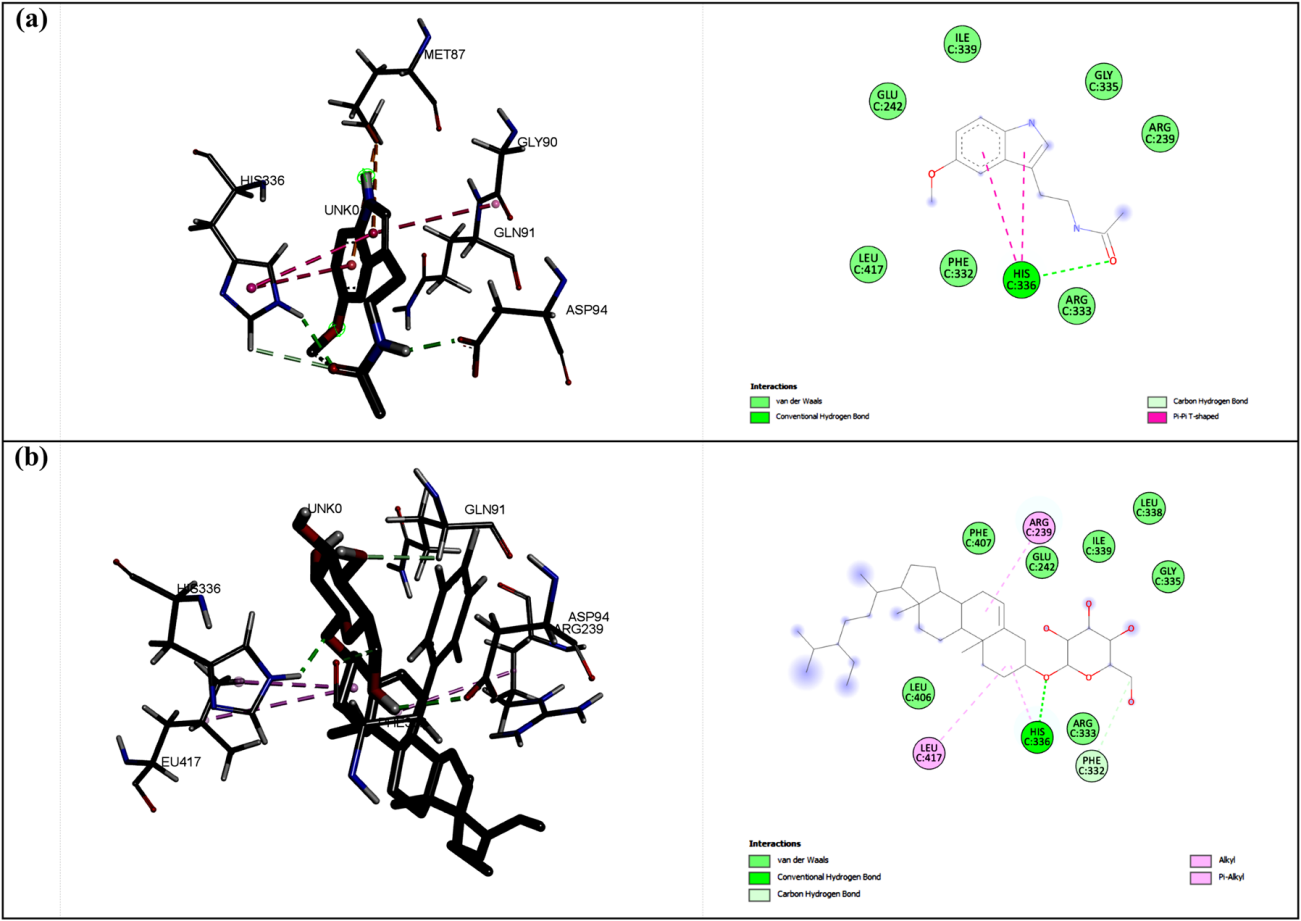
The interactions between melatonin (a) and sitogluside (b) with MP protein.

Hydrogen bonds became the most studied interaction in molecular docking and MD simulation since they had the most vital energy among the non-covalent interaction between protein and ligand [50]. In the second protein, MP, the selected ligand predicted to be responsible for the antioxidant activity was sitogluside (CID: 5742590). This ligand interacted in hydrogen bond mode with the protein via HIS336 residue, similar to the melatonin as a positive control (Figure 2). Interestingly, the binding affinity of the MP-sitogluside complex (−9.0 kcal/mol) was more substantial than MP-melatonin (−7.4 kcal/mol). This result is due to other interactions in the MP-sitogluside complex, which strengthen the binding affinity; they were alkyl and pi-alkyl interactions.

**Figure 3:**
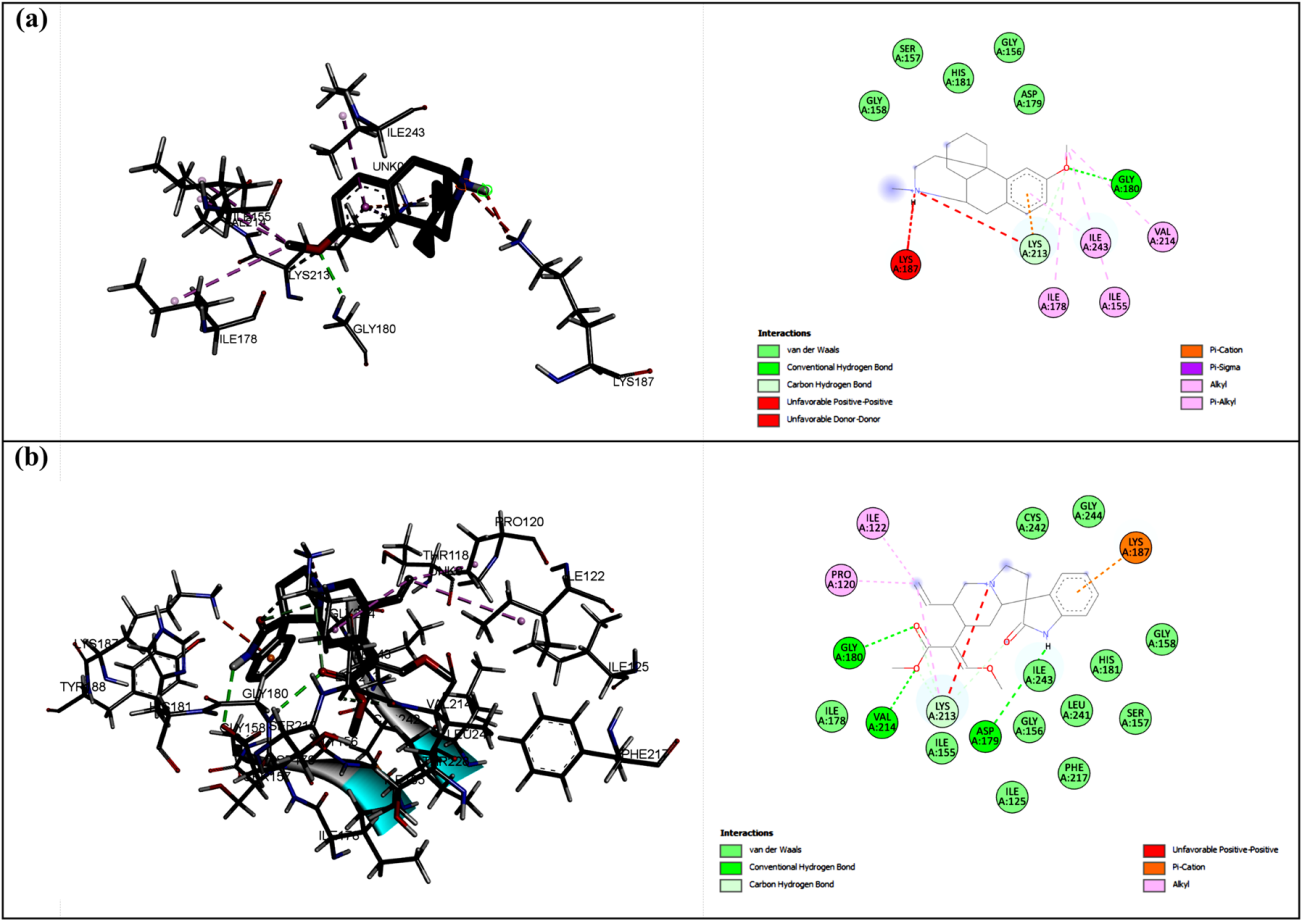
The interactions between dextromethorphan (a) and corynoxeine (b) with NO protein.

The third studied protein was NO, which had GLY180 as a primary interacting residue, according to the docking result against dextromethorphan. Based on this fact, the selected ligand from the virtual screening was corynoxeine (CID: 44568160) since this ligand was shown a hydrogen bond to GLY180 and the ASP179 and VAL214, as shown in Figure 3. Corynoxeine had more hydrogen bonds than dextromethorphan which implicated the stronger binding affinity (−7.4 kcal/mol compared to −6.3 kcal/mol). Therefore, overall, we selected ligands that exhibit a strong binding affinity and identical interactions compared to the positive control from the docking procedure. The parameter we used to choose these ligands was the binding affinity since this value corresponded to the inhibition constant (Ki). Furthermore, we also identified the hydrogen bonds between the ligand and the protein, as pharmacologically, similar bioactivity will show when a compound interacts with the protein similarly to the positive control. Based on these parameters, we take these three ligands, i.e. (-)-epicatechin, sitogluside, and corynoxeine, as the specific compounds interacting with proteins CP450, MP, and NO, respectively. To ensure the stability of the protein-ligand complexes, they were subjected to the next step in molecular dynamics simulation.

### 3.2 Frontier molecular orbital profile

**Figure 4:**
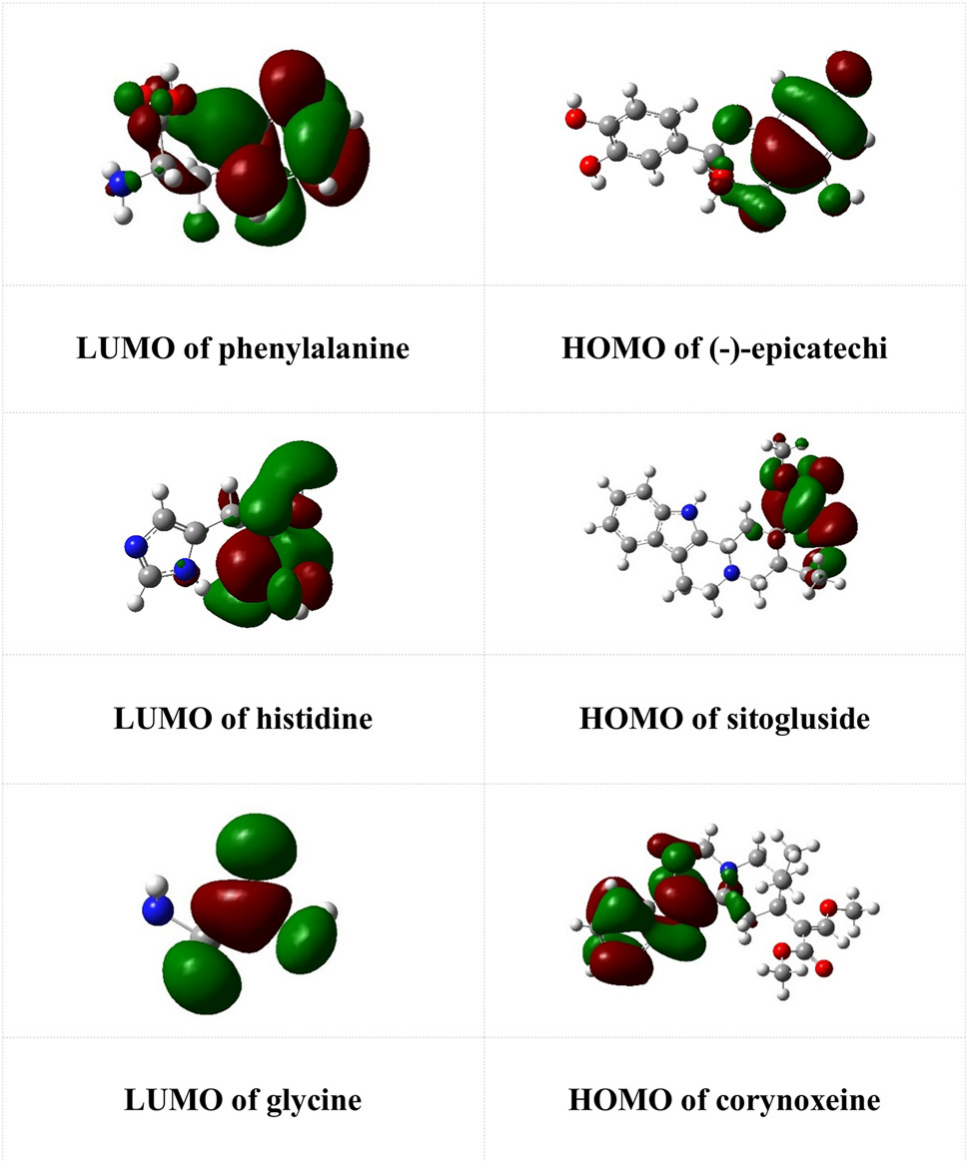
The frontier molecular orbitals of the proteins’ primary residues and selected ligands.

Since it was sentenced that the interacting molecular orbital in intermolecular interaction was between the LUMO of the protein and the HOMO of the ligand [18], we conducted a study of the frontier molecular orbitals (MOs) of the primary residues of the proteins and the selected ligands. The profile of those MOs is shown in Figure 4. The energy gap between HOMO and LUMO was about 5–6 eV, which is small enough for the electron from the HOMO of the ligand to interact with the LUMO of the targeted protein and was stable [51].

Figure 4 showed that the LUMO orbital in phenylalanine was located in the phenyl moiety, while in epicatechin, the HOMO was located in ring A of the flavonoid. In histidine, the LUMO was concentrated around the zwitterion part of the amino acid, and the HOMO of sitogluside was located in the cyclohexane attached to three hydroxyl groups. The LUMO of glycine was observed around the amino acid’s carboxyl part, while the HOMO of corynoxeine was concentrated in its amide part. The hydrogen bond interaction occurred between the HOMO of the ligand (as the donor) and the LUMO of the amino acid of the protein (as the acceptor), as reported by previous similar studies 52], [53], [54], [55.

### 3.3 ADMET properties

The result of ADMET prediction is shown in Table 2. The three compounds were predicted to have good drug-ability and were not toxic. Sitogluside was predicted to have a low gastrointestinal (GI) absorption, while the other two compounds were predicted to have a high one. The prediction resulted in low GI absorption due to the ratio between the calculated lipophilicity (WLOGP) and surface area (TPSA) being relatively high [56]. On the contrary, corynantheidine was predicted as the BBB permeant, which can permeate the brain. Fortunately, the three selected compounds were not inhibiting the CYP isoenzyme. If a compound inhibits the isoenzyme, it can cause the reduced clearance and buildup of the compound or its metabolites, resulting in toxic or other unfavorable side effects [57].

**Table 2:** The result of ADMET prediction.

Parameter	Prediction result		
	Sitogluside	Corynantheidine	(-)-Epicatechin
GI absorption	Low	High	High
BBB permeant	No	Yes	No
CYP inhibitor	No	No	No
Lipinski #violation	1	0	0
Leadlikeness #violations	3	1	0
Bioavailability score	0.55	0.55	0.55
PAINS #alerts	0	1	1
Synthetic accessibility	8.02	4.27	3.50
Predicted LD_50_	8,000 mg/kg	300 mg/kg	10,000 mg/kg
Predicted toxicity class	6	3	6
Hepatotoxicity	Inactive	Inactive	Inactive
Carcinogenicity	Inactive	Inactive	Inactive
Immunotoxicity	Active	Inactive	Inactive
Mutagenicity	Inactive	Inactive	Inactive
Cytotoxicity	Inactive	Inactive	Inactive
Tox21-nuclear receptor signaling pathways	Inactive	Inactive	Active
Tox21-stress response pathways	Inactive	Inactive	Inactive

As a pioneer in defining drug-likeness, Lipinski’s rule of five is still considered in drug discovery today. The rules were limiting the value of the molecular weight, log P, hydrogen bond donor, and hydrogen bond acceptor of a compound to have an excellent oral administration [58]. Among the three compounds, only sitogluside was predicted to violate one of the rules. However, it still can be concluded to obey Lipinski’s rules and could be approved by FDA. Moreover, it was found that some of the approved drugs violated one or two of the rules [59]. Slightly different from Lipinski, Brenk et al. define the other limitations for a compound to consider as a good lead in drug discovery [60]. In this case, sitogluside violated three rules, including more than 27 heavy atoms, more than eight rotatable bonds, and a value of ClogP/ClogD of more than 4. This fact suggests that sitogluside was not favorable to be derivated. While corynantheidine only violates one rule, and even (-)-epicatechin did not violate the rule; hence the two compounds were favorable to be lead compound.

The bioavailability score of the three compounds was calculated as 0.55. This number represents that the compounds were predicted to pass Lipinski’s rule of five, while below the number were considered to fail the rules [61]. Meanwhile, sitogluside was predicted to not shows the PAINS alert. However, another two compounds were shown one PAINS alert. This alert was related to the potency of the compound to be reactive in the human body, represented by the number of zero to six in the range [62]. The last ADME property predicted was synthetic accessibility, which shows that sitogluside was the most challenging compound to be synthesized among the three compounds, and (-)-epicatechin was the most accessible compound [63].

In terms of predicted toxicities, only corynantheidine tends to be toxic. This was represented by the number of predicted LD_50_ (300 mg/kg) and the class of toxicity (class 3). Corynantheidine can be made into the related derivative compounds to cope with this. In general, the predicted organ toxicities of the three compounds showed inactivity, except for the immunotoxicity of sitogluside and the receptor signaling pathways of (-)-epicatechin. Hence, it can be stated that all three compounds are non-toxic.

### 3.4 MD simulation

**Figure 5:**
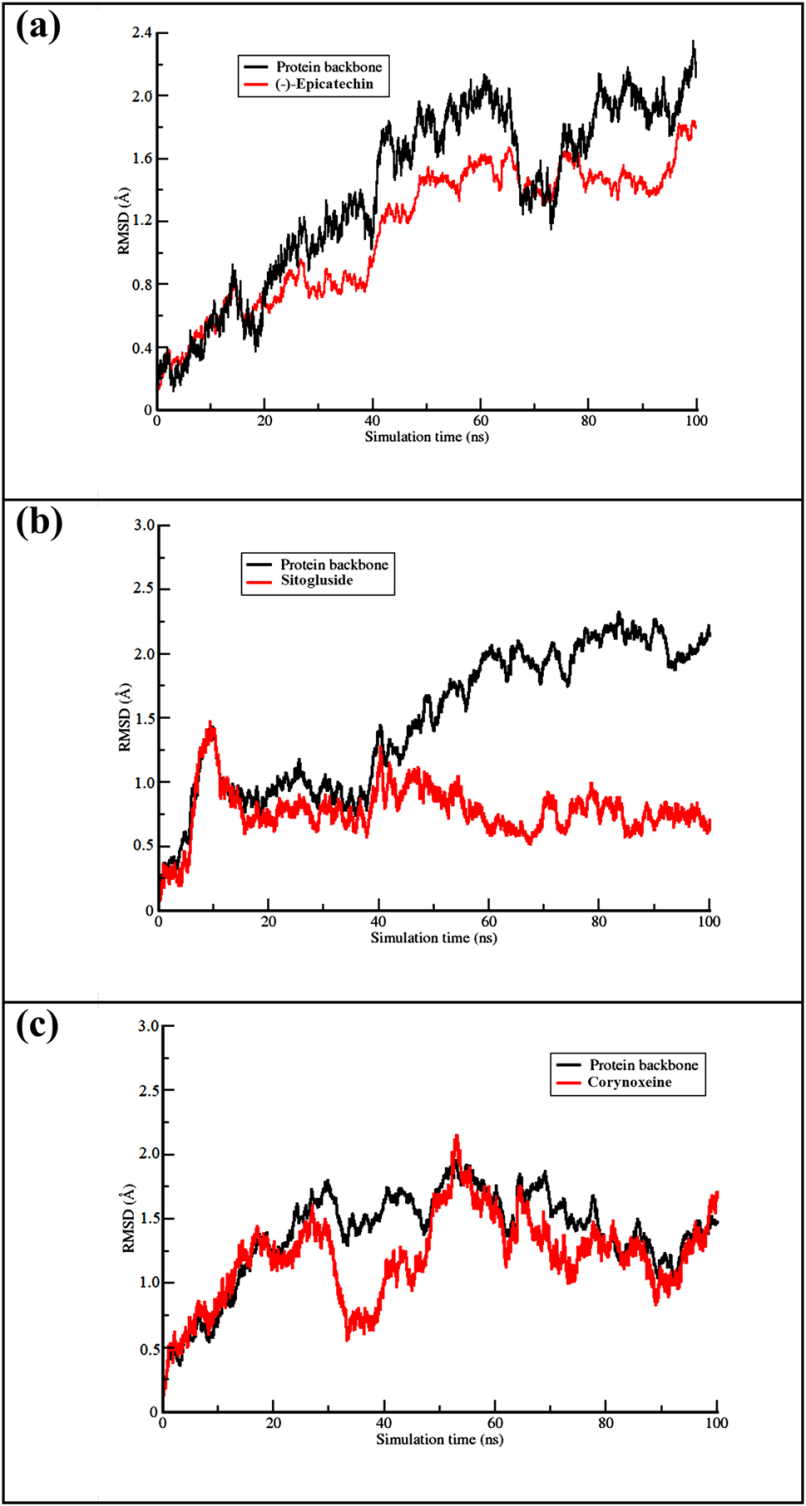
The RMSD profile of CP450-(-)-epicathecin (a), MP-sitogluside (b), and NO-corynoxeine (c) complex.

Due to the existence of water molecules and the protein being set to be flexible during simulation, molecular dynamics (MD) represents a more similar natural situation than molecular docking. Figure 5 shows the results of the RMSD profiles of protein and ligand backbone studied in this work using the *cpptraj* function [64]. Based on the profiles, the sitogluside-CP450 and corynantheidine-MP complexes showed stability after 25 ns. While the (-)-epicatechin shows more stability than the NO as its targeted protein, which indicates the ligand interacted with the protein’s active site. This phenomenon was in line with our SBVS result in Table 1 which shows that the binding affinity of the complex was relatively strong.

**Figure 6:**
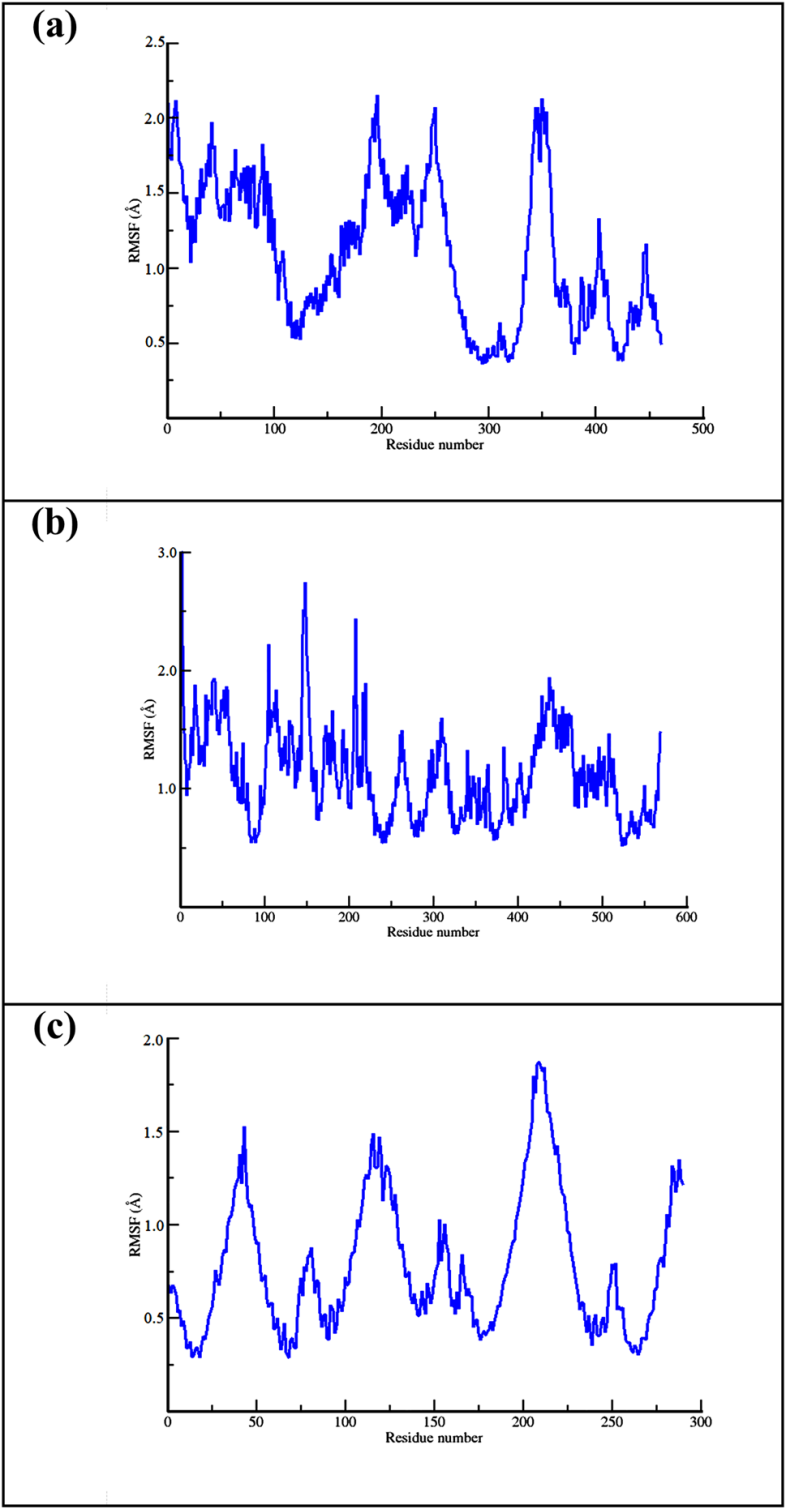
The RMSF profile of CP450 -(-)-epicathecin (a), MP-sitogluside (b), and NO-corynoxeine (c) complex.

If the RMSD shows the fluctuation of atoms during the simulation time, the RMSF shows the fluctuation of a particular protein residue on average. The result is shown in Figure 6. Generally, the residues of the three proteins still fluctuated within an acceptable range of RMSF, between 1 and 3 Å [65]. Among the three proteins, NO showed the most fluctuating one since the residues with a number around 200 have an RMSF value reaching 3 Å.

We calculate the RMSF to ensure the primary interacting residue(s) in each protein was not fluctuating more than 2 Å (the maximum length of hydrogen bond). In CP450, the primary residue, GLY90 and PHE100, had around 1 Å of fluctuation. The same phenomenon was observed in the MP protein, where HIS336 as the primary residue fluctuated below 2 Å. The last protein, NO, which has GLY180, ASP179, and VAL214 as the primary interacting residues, shows low fluctuation for those three primary residues.

**Figure 7:**
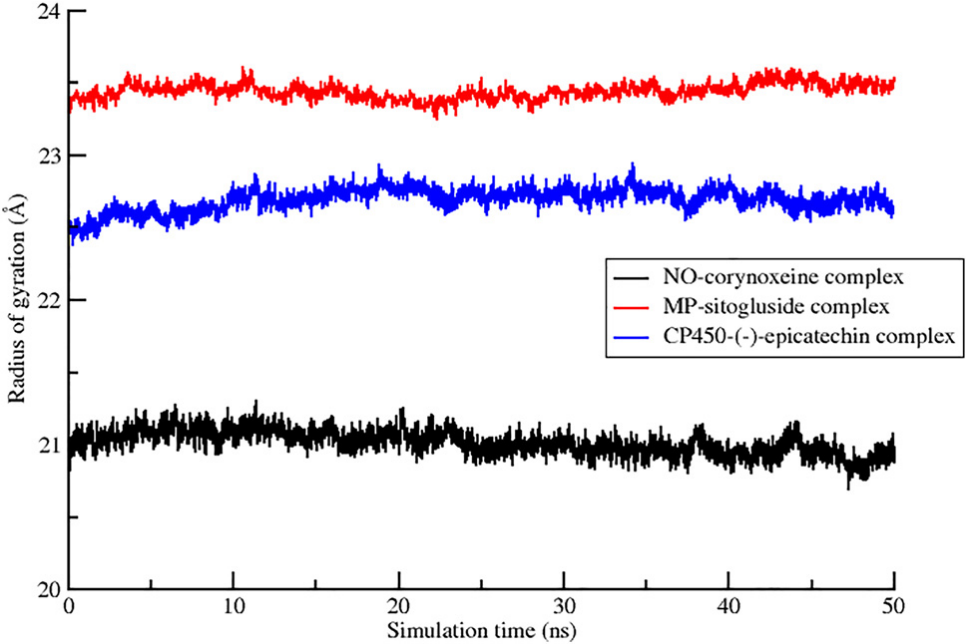
The radius of gyration of the CP450-(-)-epicathecin, MP-sitogluside, and NO-corynoxeine complexes.

Furthermore, the RoG data in Figure 7 were not intended for a direct comparison of compactness across the three complexes. Instead, this RoG shows each complex exhibited a relatively stationary pattern of RoG values throughout the simulation period which indicate a stable conformation without significant structural changes. It is also important to note that the RoG values are inherently influenced by the number of residues in each protein. For instance, the MP complex, which comprises 578 residues, exhibited the largest RoG value among the three complexes. In contrast, the CP450 and NO proteins, containing 490 and 451 residues respectively, showed smaller RoG values.

As the most studied interaction in molecular modeling, hydrogen bonds are essential in determining the protein-ligand complexes’ stability. Table 3 shows the result of the hydrogen bond analysis from the three complexes in this work. The MD results confirmed the stability of molecular docking-produced complexes, as the hydrogen bonds which occurred in the previous methods were also observed in the MD results.

**Table 3:** Hydrogen bond analysis result.

System	Acceptor	DonorH	Donor	Occupancy (%)	Average distance
CP450 complex	PHE_98@O	LIG_462@H12	LIG_462@O4	12.8	1.0128
	GLY_100@OD2	LIG_462@H14	LIG_462@O6	9.2	1.0092
	LIG_462@O5	ALA_268@H	ALA_268@N	0.4	1.0004
	GLY_69@O	LIG_462@H11	LIG_462@O3	0.2	1.0002
	LIG_462@O6	ALA_268@H	ALA_268@N	0.2	1.0002
	PRO_338@N	LIG_462@H12	LIG_462@O4	0.2	1.0002
	ALA_77@O	LIG_462@H14	LIG_462@O6	0.2	1.0002
MP complex	MET_87@O	LIG_570@H11	LIG_570@O3	7.4	1.0089
	HIS_336@O	LIG_570@H12	LIG_570@O4	6.8	1.0082
	GLY_326@O	LIG_570@H11	LIG_570@O3	0.6	1.0007
	GLY_326@N	LIG_570@H12	LIG_570@O4	0.2	1.0002
	ASP_94@OD1	LIG_570@H12	LIG_570@O4	0.2	1.0002
	ILE_327@NE2	LIG_570@H12	LIG_570@O4	0.2	1.0002
NO complex	ASP_178@OD2	LIG_451@H1	LIG_451@N2	56.9	1.7113
	ASP_178@OD1	LIG_451@H1	LIG_451@N2	19.58	1.2447
	LIG_451@O1	GLY_179@HZ3	GLY_179@NZ	5.04	1.0630
	LIG_451@O1	LYS_212@HZ1	LYS_212@NZ	5.02	1.0628
	LIG_451@O1	LYS_212@HZ2	LYS_212@NZ	4.34	1.0542
	LIG_451@N1	LYS_212@HZ3	LYS_212@NZ	1.42	1.0177
	LIG_451@O3	VAL_213@H	VAL_213@N	1.38	1.0173
	LIG_451@N1	LYS_212@HZ2	LYS_212@NZ	1.12	1.0140
	LIG_451@N1	LYS_212@HZ1	LYS_212@NZ	1.02	1.0127
	LIG_451@O2	VAL_213@H	VAL_213@N	0.74	1.0092
	LIG_451@O2	GLY_179@H	GLY_179@N	0.06	1.0008
	LIG_451@O2	LYS_212@HZ1	LYS_212@NZ	0.02	1.0003
	LIG_451@O2	LYS_212@HZ3	LYS_212@NZ	0.02	1.0003

*LIG was the selected ligand for each of the protein.

In the first complex, the hydrogen bond between ligand ((-)-Epicatechin) and PHE98 was the most lasting, while the second interaction between the ligand and GLY100 lasted for a slightly shorter time. In the MP complex, the primary interaction was not the longest-lasting hydrogen bond. However, the hydrogen bond between the ligand and HIS336 had an occupancy of 6.8 %, which was still significant. Interestingly, the hydrogen bonds formed between ligand and NO had the relatively highest occupancy, especially to the primary residues (ASP178 and GLY179). This result agreed with the RMSD profile of the ligand shown in Figure 5c, which shows the flattening curve. Regarding interaction distance, all of the hydrogen bonds had a short distance, around 1 Å, suggesting that the interactions between the proteins and ligands are relatively strong [66]. However, the distance of the hydrogen bond between the oxygen of ASP178 from protein NO and hydrogen from (-)-epicatechin was relatively long compared to the others observed in the systems.

The MMPBSA methods provided more detailed information on the energies involved in protein-ligand interactions, making it helpful in rescoring virtual screening results 67], [68], [69. Table 4 shows the result of free-energy binding estimation. The van der Waals energy and dispersion energy contributions in the three systems were around 40–50 kcal/mol, while the PB energy was similar for the CP450 and MP but not for the NO. The value of PB energy above 40 kcal/mol shows that the solvent contribution in this system was dominant since the PB term was calculating the electrostatic contribution to the solvation energy [46, 70]. Interestingly, no electrostatic solvation contribution is observed in CP450 and MP complexes, while in NO, this term was calculated in a relatively remarkable value. The nonpolar energy was similar among the three systems, which consists of cavitation and repulsion energies [71]. The NO complex has the weakest value on the total Gibbs free energy, while Table 3 shows more hydrogen bonds than the other two complexes. This result is consistent with the statement by Klebe and Bohm that the number of hydrogen bonds is not always related to the strength of the interaction [72].

**Table 4:** The components of free energy calculated by MMPBSA.

System	Energy (kcal/mole)							
	Δ*E*_vdw_	Δ*E*_El_	Δ*E*_PB_	Δ*E*_NPol_	Δ*E*_Disp_	Δ*G*_gas_	Δ*G*_solv_	Δ*G*_total_
CP450 complex	−43.20	0	11.08	−22.91	47.42	−43.20	35.58	−7.61
MP complex	−52.35	0	12.88	−24.37	49.39	−52.35	37.91	−14.44
NO complex	−40.27	−23.39	42.96	−30.36	49.95	−63.66	62.55	−1.10

Δ*E*_vdw_: the van der Waals energies; Δ*E*_PB_: the Poisson-Boltzmann energies; Δ*E*_NPol_: the nonpolar contribution to the solvation energies; Δ*E*_Disp_: the dispersion energy; Δ*G*_gas_: the sum of Δ*E*_vdw_ + Δ*E*_El_; Δ*G*_solv_: the sum of Δ*E*_PB_ + Δ*E*_NPol_ + Δ*E*_Disp_; Δ*G*_total_: the sum of Δ*G*_gas_ + Δ*G*_solv_.

This study successfully identified the compounds responsible for the antioxidant activity found in *M. speciosa* leaves. The compounds (-)-epicatechin, sitogluside, and corynoxeine have been seen as the specific compounds that interact with proteins CP450, MP, and NO, respectively. The results obtained from this computational study provide a solid foundation for future *in vitro* tests, assisting experimental researchers in validating the antioxidant activity using these three most promising compounds.

## 4 Conclusions

This study demonstrates that there are several compounds responsible for the antioxidant activity of *M. speciosa* leaves. The compounds were (-)-epicatechin, sitogluside, and corynoxeine to each targeted protein, namely CP450, MP, and NO. The frontier molecular orbital profiles show the correlating explanation of the interaction between the ligands and their targeted proteins. The molecular dynamics simulation confirmed the stability of the protein-ligand complexes by the profile of RMSD, RMSF, RoG, and hydrogen bonds. We expect our work to guide further experimental studies on the *in vitro* test to assess the antioxidant properties of the three compounds identified in this computational study.

## References

[1] Dharmaraja AT (2017). Role of reactive oxygen species (ROS) in therapeutics and drug resistance in cancer and bacteria. J Med Chem.

[2] Veith A, Moorthy B (2018). Role of cytochrome P450s in the generation and metabolism of reactive oxygen species. Curr Opin Toxicol.

[3] Chen S, Chen H, Du Q, Shen J (2020). Targeting myeloperoxidase (MPO) mediated oxidative stress and inflammation for reducing brain ischemia injury: potential application of natural compounds. Front Physiol.

[4] Tarafdar A, Pula G (2018). The role of NADPH oxidases and oxidative stress in neurodegenerative disorders. Int J Mol Sci.

[5] Youssef D, El-Bakatoushi R, Elframawy A, El-Sadek L, El Badan G (2023). Molecular phylogenetic study of flavonoids in medicinal plants: a case study family Apiaceae. J Plant Res.

[6] (2022). US Association initiates partnership in kratom export with Indonesia. https://ksp.go.id/en/us-association-initiates-partnership-in-kratom-export-with-indonesia.

[7] Firmansyah A, Sundalian M, Taufiq M (2020). Kratom (Mitragyna speciosa Korth) for a new medicinal: a review of pharmacological and compound analysis. Biointerface Res Appl Chem.

[8] Flores-Bocanegra L, Raja HA, Graf TN, Augustinović M, Wallace ED, Hematian S (2020). The chemistry of kratom [Mitragyna speciosa]: updated characterization data and methods to elucidate indole and oxindole alkaloids. J Nat Prod.

[9] Chear NJY, León F, Sharma A, Kanumuri SRR, Zwolinski G, Abboud KA (2021). Exploring the chemistry of alkaloids from Malaysian Mitragyna speciosa (kratom) and the role of oxindoles on human opioid receptors. J Nat Prod.

[10] Boulaamane Y, Ibrahim MAA, Britel MR, Maurady A (2022). *In silico* studies of natural product-like caffeine derivatives as potential MAO-B inhibitors/AA 2A R antagonists for the treatment of Parkinson’s disease. J Integr Bioinform.

[11] List M (2017). Using docker compose for the simple deployment of an integrated drug target screening platform. J Integr Bioinform.

[12] Brunak S, Bjerre Collin C, Eva Ó Cathaoir K, Golebiewski M, Kirschner M, Kockum I (2020). Towards standardization guidelines for *in silico* approaches in personalized medicine. J Integr Bioinform.

[13] Varela-Rial A, Majewski M, De Fabritiis G (2022). Structure based virtual screening: fast and slow. Wiley Interdiscip Rev Comput Mol Sci.

[14] Arief I, Kurnianto E (2022). Identification of active compound from Mitragyna speciosa leave as antiinflammation agent: *in silico* study. Acta Chim Asiana.

[15] Sabe VT, Ntombela T, Jhamba LA, Maguire GE, Govender T, Naicker T (2021). Current trends in computer aided drug design and a highlight of drugs discovered via computational techniques: a review. Eur J Med Chem.

[16] Boufissiou A, Abdalla M, Sharaf M, Al-Resayes SI, Imededdine K, Alam M (2022). *In-silico* investigation of phenolic compounds from leaves of Phillyrea angustifolia L. as a potential inhibitor against the SARS-CoV-2 main protease (Mpro PDB ID:5R83) using a virtual screening method. J Saudi Chem Soc.

[17] Albratty M (2022). Quantitative structure–activity relationship modeling and docking of some synthesized bioactive oxopyrolidines against Staphylococcus aureus. J Saudi Chem Soc.

[18] Pang X, Zhou L, Zhang M, Zhang L, Xu L, Xie F (2014). Two rules on the protein-ligand interaction. Open Conf Proc J.

[19] Rahman M, Talukder A, Akter R (2021). Computational designing and prediction of ADMET properties of four novel imidazole-based drug candidates inhibiting heme oxygenase-1 causing cancers. Mol Inf.

[20] Kar S, Leszczynski J (2020). Open access *in silico* tools to predict the ADMET profiling of drug candidates. Expert Opin Drug Discov.

[21] Jia CY, Li JY, Hao GF, Yang GF (2020). A drug-likeness toolbox facilitates ADMET study in drug discovery. Drug Discov Today.

[22] Dimić D, Milanović Ž, Jovanović G, Sretenović D, Milenković D, Marković Z (2020). Comparative antiradical activity and molecular docking/dynamics analysis of octopamine and norepinephrine: the role of OH groups. Comput Biol Chem.

[23] Jamali T, Kavoosi G, Jamali Y, Mortezazadeh S, Ardestani SK (2021). *In-vitro*, *in-vivo*, and *in-silico* assessment of radical scavenging and cytotoxic activities of Oliveria decumbens essential oil and its main components. Sci Rep.

[24] de Almeida VM, Dias ÊR, de Souza BC, Leite FHA, Biondi I, Vieira IJC (2023). Myeloperoxidase inhibition and *in silico* evaluation of Phenolics from Vellozia dasypus. Rev Bras Farmacogn.

[25] Salaria D, Rolta R, Sharma N, Patel CN, Ghosh A, Dev K (2022). *In vitro* and *in silico* antioxidant and anti-inflammatory potential of essential oil of Cymbopogon citratus (DC.) Stapf. of North-Western Himalaya. J Biomol Struct Dyn.

[26] Saurabh S, Sivakumar PM, Perumal V, Khosravi A, Sugumaran A, Prabhawathi V (2020). Molecular dynamics simulations in drug discovery and drug delivery. Eng Mater.

[27] Adelusi TI, Oyedele AQK, Boyenle ID, Ogunlana AT, Adeyemi RO, Ukachi CD (2022). Molecular modeling in drug discovery. Inform Med Unlocked.

[28] Kato K, Nakayoshi T, Fukuyoshi S, Kurimoto E, Oda A (2017). Validation of molecular dynamics simulations for prediction of three-dimensional structures of small proteins. Molecules.

[29] Merz PT, Shirts MR (2018). Testing for physical validity in molecular simulations. PLoS One.

[30] van Gunsteren WF, Daura X, Hansen N, Mark AE, Oostenbrink C, Riniker S (2018). Validation of molecular simulation: an overview of issues. Angew Chem – Int Ed.

[31] Pettersen EF, Goddard TD, Huang CC, Couch GS, Greenblatt DM, Meng EC (2004). UCSF Chimera: a visualization system for exploratory research and analysis. J Comput Chem.

[32] Gasteiger J, Marsili M (1978). A new model for calculating atomic charges in molecules. Tetrahedron Lett.

[33] Jakalian A, Bush BL, Jack DB, Bayly CI (2000). Fast, efficient generation of high-quality atomic charges. AM1-BCC model: I. Method. J Comput Chem.

[34] Wang J, Wang W, Kollman PA, Case DA (2006). Automatic atom type and bond type perception in molecular mechanical calculations. J Mol Graph Model.

[35] Trott O, Olson AJ (2009). AutoDock Vina: improving the speed and accuracy of docking with a new scoring function, efficient optimization, and multithreading. J Comput Chem.

[36] Dallakyan S, Olson AJ (2015). Small-molecule library screening by docking with PyRx. Methods Mol Biol.

[37] Costa J, Ramos RDS, Costa KDSL, Brasil DDSB, Silva CHTDPD, Ferreira EFB (2018). An *in silico* study of the antioxidant ability for two caffeine analogs using molecular docking and quantum chemical methods. Molecules.

[38] BIOVIA (2021). Dassault Systèmes, Discovery Studio Visualizer, v21.1.0.20298.

[39] Frisch MJ, Trucks GW, Schlegel HB, Scuseria GE, Robb MA, Cheeseman JR (2016). Gaussian 16, Revision C.01.

[40] Hagar M, Ahmed HA, Aljohani G, Alhaddad OA (2020). Investigation of some antiviral N-heterocycles as COVID 19 drug: molecular docking and DFT calculations. Int J Mol Sci.

[41] Daina A, Michielin O, Zoete V (2017). SwissADME: a free web tool to evaluate pharmacokinetics, drug-likeness and medicinal chemistry friendliness of small molecules. Sci Rep.

[42] Banerjee P, Eckert AO, Schrey AK, Preissner R (2018). ProTox-II: a webserver for the prediction of toxicity of chemicals. Nucleic Acids Res.

[43] Case DA, Aktulga HM, Belfon KAA, Ben-Shalom I, Berryman JT, Brozell SR (2022). Amber 2022.

[44] Case DA, Cheatham TE, Darden T, Gohlke H, Luo R, Merz KM (2005). The Amber biomolecular simulation programs. J Comput Chem.

[45] Mark P, Nilsson L (2001). Structure and dynamics of the TIP3P, SPC, and SPC/E water models at 298 K. J Phys Chem A.

[46] Miller BR, McGee TD, Swails JM, Homeyer N, Gohlke H, Roitberg AE (2012). MMPBSA.py : an efficient program for end-state free energy calculations. J Chem Theor Comput.

[47] Vaish S, Parveen R, Rajneesh, Singh N, Gupta D, Basantani MK (2022). Computational insights into diverse aspects of glutathione S-transferase gene family in Papaver somniferum. J Plant Res.

[48] Williams PA, Cosme J, Ward A, Angove HC, Vinković DM, Jhoti H (2003). Crystal structure of human cytochrome P450 2C9 with bound warfarin. Nature.

[49] Huang J, Smith F, Panizzi JR, Goodwin DC, Panizzi P (2015). Inactivation of myeloperoxidase by benzoic acid hydrazide. Arch Biochem Biophys.

[50] Salentin S, Haupt VJ, Daminelli S, Schroeder M (2014). Polypharmacology rescored: protein–ligand interaction profiles for remote binding site similarity assessment. Prog Biophys Mol Biol.

[51] Rahuman MH, Muthu S, Raajaraman BR, Raja M, Umamahesvari H (2020). Investigations on 2-(4-cyanophenylamino) acetic acid by FT-IR,FT-Raman, NMR and UV-Vis spectroscopy, DFT (NBO, HOMO-LUMO, MEP and Fukui function) and molecular docking studies. Heliyon.

[52] Balajee R, Srinivasadesikan V, Sakthivadivel M, Gunasekaran P (2016). *In silico* screening, alanine mutation, and DFT approaches for identification of NS2B/NS3 protease inhibitors. Biochem Res Int.

[53] Banavath HN, Sharma OP, Kumar MS, Baskaran R (2014). Identification of novel tyrosine kinase inhibitors for drug resistant T315I mutant BCR-ABL: a virtual screening and molecular dynamics simulations study. Sci Rep.

[54] Nagamani S, Muthusamy K (2018). A theoretical insight to understand the molecular mechanism of dual target ligand CTA-018 in the chronic kidney disease pathogenesis. PLoS One.

[55] Shanmugam G, Lee S, Jeon J (2018). Identification of potential nematicidal compounds against the pine wood nematode, Bursaphelenchus xylophilus through an *in silico* approach. Molecules.

[56] Daina A, Zoete V (2016). A BOILED-egg to predict gastrointestinal absorption and brain penetration of small molecules. ChemMedChem.

[57] Kirchmair J, Göller AH, Lang D, Kunze J, Testa B, Wilson ID (2015). Predicting drug metabolism: experiment and/or computation?. Nat Rev Drug Discov.

[58] Lipinski CA, Lombardo F, Dominy BW, Feeney PJ (1997). Experimental and computational approaches to estimate solubility and permeability in drug discovery and development settings. Adv Drug Deliv Rev.

[59] Shultz MD (2019). Two decades under the influence of the rule of five and the changing properties of approved oral drugs. J Med Chem.

[60] Brenk R, Schipani A, James D, Krasowski A, Gilbert I, Frearson J (2008). Lessons learnt from assembling screening libraries for drug discovery for neglected diseases. ChemMedChem.

[61] Martin YC (2005). A bioavailability score. J Med Chem.

[62] Baell JB, Holloway GA (2010). New substructure filters for removal of pan assay interference compounds (PAINS) from screening libraries and for their exclusion in bioassays. J Med Chem.

[63] Ertl P, Schuffenhauer A (2009). Estimation of synthetic accessibility score of drug-like molecules based on molecular complexity and fragment contributions. J Cheminf.

[64] Roe DR, Cheatham TE (2013). PTRAJ and CPPTRAJ: software for processing and analysis of molecular dynamics trajectory data. J Chem Theor Comput.

[65] Khanna V, Ranganathan S, Petrovsky N (2018). Rational structure-based drug design.

[66] Herschlag D, Pinney MM (2018). Hydrogen bonds: simple after all?. Biochemistry.

[67] Sahakyan H (2021). Improving virtual screening results with MM/GBSA and MM/PBSA rescoring. J Comput Aided Mol Des.

[68] Hu X, Contini A (2019). Rescoring virtual screening results with the MM-PBSA methods: beware of internal dielectric constants. J Chem Inf Model.

[69] Wang E, Sun H, Wang J, Wang Z, Liu H, Zhang JZH (2019). End-point binding free energy calculation with MM/PBSA and MM/GBSA: strategies and applications in drug design. Chem Rev.

[70] Srinivasan J, Cheatham TE, Cieplak P, Kollman PA, Case DA (1998). Continuum solvent studies of the stability of DNA, RNA, and phosphoramidate-DNA helices. J Am Chem Soc.

[71] Genheden S, Ryde U (2015). The MM/PBSA and MM/GBSA methods to estimate ligand-binding affinities. Expert Opin Drug Discov.

[72] Klebe G, Böhm H-J (1997). Energetic and entropic factors determining binding affinity in protein-ligand complexes. J Recept Signal Transduction.

